# Characterization of the Mitochondrial Genome of a Wheat AL-Type Male Sterility Line and the Candidate CMS Gene

**DOI:** 10.3390/ijms22126388

**Published:** 2021-06-15

**Authors:** Miaomiao Hao, Wenlong Yang, Weiwen Lu, Linhe Sun, Muhammad Shoaib, Jiazhu Sun, Dongcheng Liu, Xin Li, Aimin Zhang

**Affiliations:** 1 State Key Laboratory of Plant Cell and Chromosome Engineering, Institute of Genetics and Developmental Biology, Innovative Academy of Seed Design, Chinese Academy of Sciences, Beijing 100101, China; mmhao@genetics.ac.cn (M.H.); weiwen.lu@genetics.ac.cn (W.L.); knight9001@163.com (L.S.); shoaib.cas@hotmail.com (M.S.); jzsun@genetics.ac.cn (J.S.); dcliu@genetics.ac.cn (D.L.); lixin@genetics.ac.cn (X.L.); 2 University of Chinese Academy of Sciences, Beijing 100049, China; 3 Institute of Vegetables and Flowers, Chinese Academy of Agricultural Sciences, Beijing 100081, China; 4 Institute of Botany, Jiangsu Province and Chinese Academy of Sciences (Nanjing Botanical Garden Memorial Sun Yat-Sen), Nanjing 210014, China

**Keywords:** AL18A, T-CMS, tapetum PCD, mitochondrial genome, *orf279*

## Abstract

Heterosis utilization is very important in hybrid seed production. An AL-type cytoplasmic male sterile (CMS) line has been used in wheat-hybrid seed production, but its sterility mechanism has not been explored. In the present study, we sequenced and verified the candidate CMS gene in the AL-type sterile line (AL18A) and its maintainer line (AL18B). In the late uni-nucleate stage, the tapetum cells of AL18A showed delayed programmed cell death (PCD) and termination of microspore at the bi-nucleate stage. As compared to AL18B, the AL18A line produced 100% aborted pollens. The mitochondrial genomes of AL18A and AL18B were sequenced using the next generation sequencing such as Hiseq and PacBio. It was found that the mitochondrial genome of AL18A had 99% similarity with that of *Triticum timopheevii*, AL18B was identical to that of *Triticum aestivum* cv. Chinese Yumai. Based on transmembrane structure prediction, 12 *orfs* were selected as candidate CMS genes, including a previously suggested *orf256*. Only the lines harboring *orf279* showed sterility in the transgenic *Arabidopsis* system, indicating that *orf279* is the CMS gene in the AL-type wheat CMS lines. These results provide a theoretical basis and data support to further analyze the mechanism of AL-type cytoplasmic male sterility in wheat.

## 1. Introduction

Plant cytoplasmic male sterility (CMS) is a maternally inherited trait maintaining female fertility but producing abortive pollen. CMS is widespread among plants and widely used to produce hybrids with significant heterosis [1]. CMS is usually associated with chimeric open reading frames (ORF) caused by mitochondrial genome reorganization [2,3,4]. In many cases, pollen fertility of the CMS plants can be restored by corresponding nuclear-encoded fertility restorer genes which indicate that CMS is an ideal model system to study nuclear-cytoplasmic gene interaction [5]. Much progress has been made in the molecular mechanism of CMS and fertility restoration in plants. Many novel chimeric mitochondrial genes have been identified and are responsible for CMS through a variety of mechanisms (such as *orf79*/*orfH79/WA352* from rice, *urf13*/*orf355* from maize, *pcf* from petunia, *orf224*/*orf222* from rapeseed, etc.) [3,6,7,8,9,10,11,12,13]. The fertility restoring genes are present in the nucleus, and most of them are triangular pentapeptide repeat (PPR) proteins (such as *Rf1a* and *Rf1b* to *orf79* in rice, *Rf-PPR592* to *pcf* in petunia, etc.) [8,14].

There are three main types of CMS in wheat, i.e., CMS-T, CMS-K, and CMS-V [15]. *Orf256*, located upstream of *COXI*, might be the sterility gene in the CMS-T type plants [16,17]. Eight restorer genes (*Rf1-Rf8*) for CMS-T type have been mapped on Chromosome 1A, 1B, 2D, 5D, 6B, 6D, 7B, and 7D; and five (*Rf1*, *Rf2*, *Rf4*, *Rf5*, *Rf7*) of them originated from *T. timopheevi* [18]. *Rf1* and *Rf3* of CMS-T type can cleave the mitochondrial gene *orf279* and restore fertility in transgenic wheat [19]. There are large repetitive units in the mitochondrial genome of the CMS-K type line; however, the maintainer line is similar to the Chinese Spring wheat [20]. CMS-AL, another type of male sterility derived from the F_2_ of Bainong 71-22 × Xinong 73 (36) 9-1-16, produced 100% sterile pollens; and its restorer, Bainong 86T2026, has strong restoring ability controlled by a single dominant gene. 99AR144-1 can restore the fertility of AL18A, studies have shown one major QTL (Chr1B) and one minor QTL (Chr5A) in 99AR144-1 [21]. AL18A is an excellent sterile line with a certain angle of glume opening, stigma partly exposed, and strong vitality and long duration. The natural outcrossing rate of AL18A is 65.02%, which is good for hybrid seeds production [22]. Using the AL-type CMS line AL18A, a wheat hybrid line Xindong 43 has been produced [23]. Their cytoplasm was believed to be originated from the common wheat variety “Huixianhong”, however, the molecular genetic mechanism of this CMS line is still not clear. In the present study, we sequenced the mitochondrial genomes of AL18A and its maintainer line AL18B and identified the candidate genes for AL-type CMS in wheat. Our results provide further insight to elaborate the mechanism of AL-type CMS in wheat, and the CMS gene could be used to design molecular markers to facilitate the application of AL18A in heterosis utilization and breeding in wheat.

## 2. Results

### 2.1. The Wheat AL-CMS Line AL18A Is the Typical Sterile Type

**Figure 1:**
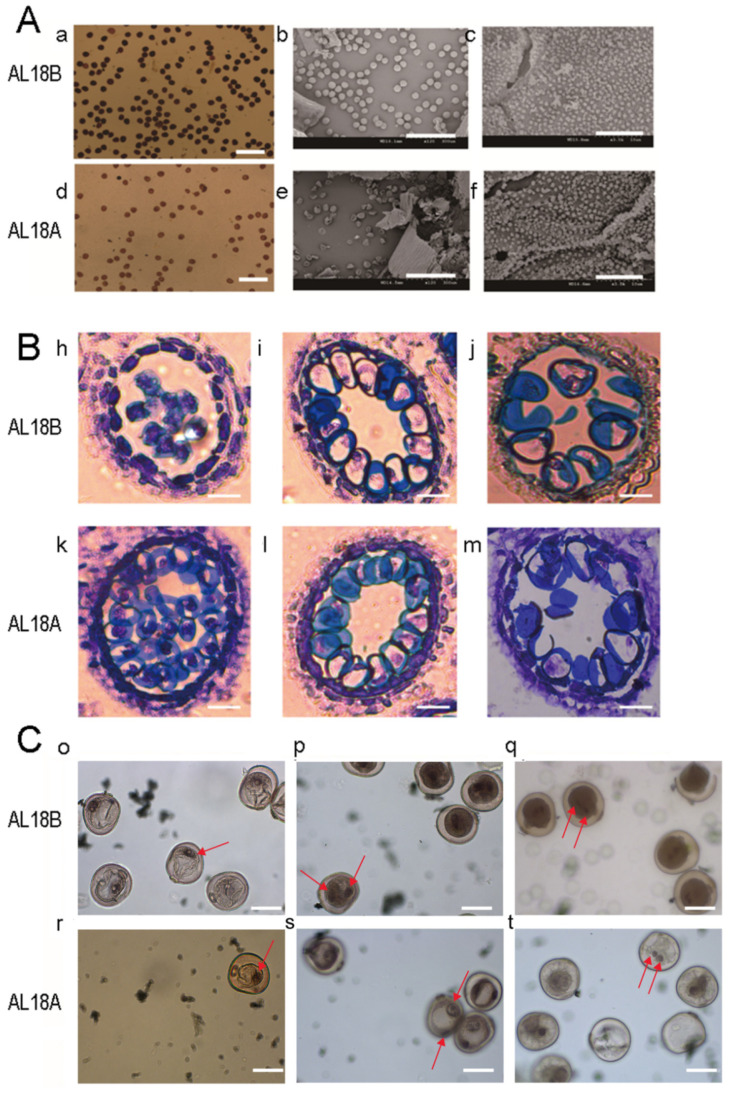
Comparison of anther and pollen grains of AL18B and AL18A. (**A**), (a,d) Mature pollen grains of AL18B and AL18A stained with I_2_-KI (Scale bar: 200 μm); (b,e) Mature pollen grains of AL18B and AL18A (Scale bar: 300 μm); (c,f) Tapetal cell surface-localized orbicule (Or) structures of AL18B and AL18A (Scale bars: 10 µm). (**B**), (h,i,j) Paraffin sections of early-uninucleate, later-uninucleate, and binucleate stage of AL18B. (k,l,m) Paraffin sections of early-uninucleate, later-uninucleate, and binucleate stage of AL18A (Scale bars: 10 µm). (**C**), (o–q) Carmine acetate dyeing of pollen in uninucleate, early-binucleate, and later- binucleate stage of AL18B. (r–t) Carmine acetate dyeing of pollen in uninucleate, early-binucleate, and later- binucleate stage of AL18A, red arrows indicate nucleus. (Scale bars: 30 µm).

The seed setting rate of the AL18A was calculated to confirm its sterility. Selfing of AL18A produced no seed; however, an AL18A × AL18B cross showed a 90% seed set (Figure S1). The pollens of AL18A were abortive, but the pistils were normal. By I_2_-KI staining, it was found that the mature pollens of AL18A were smaller and lighter as compared to that of AL18B. Scanning electron microscope (SEM) was used to examine the inner and outer walls of anthers and pollen grains from AL18A and AL18B. Results revealed that the pollen grains of AL18A were shriveled and shrunken; however, no significant difference was detected between the inner walls of the anthers from AL18A and AL18B, respectively (Figure 1A). The earliest abnormal anther of AL18A was observed through the paraffin sectioning at the monocyte vacuole stage. The tapetum cells of AL18A also had delayed cell collapse compared to AL18B (Figure 1B). The pollen grains of AL18A showed abnormal nuclear division and light color staining at the bi-nucleate stage, which might have seized its development because of which the tri- nucleate stage was not detected (Figure 1C). These results suggested that the AL18A showed a typical type of pollen abortion.

### 2.2. Mitochondrial Genome of Wheat AL18A and AL18B

**Figure 2:**
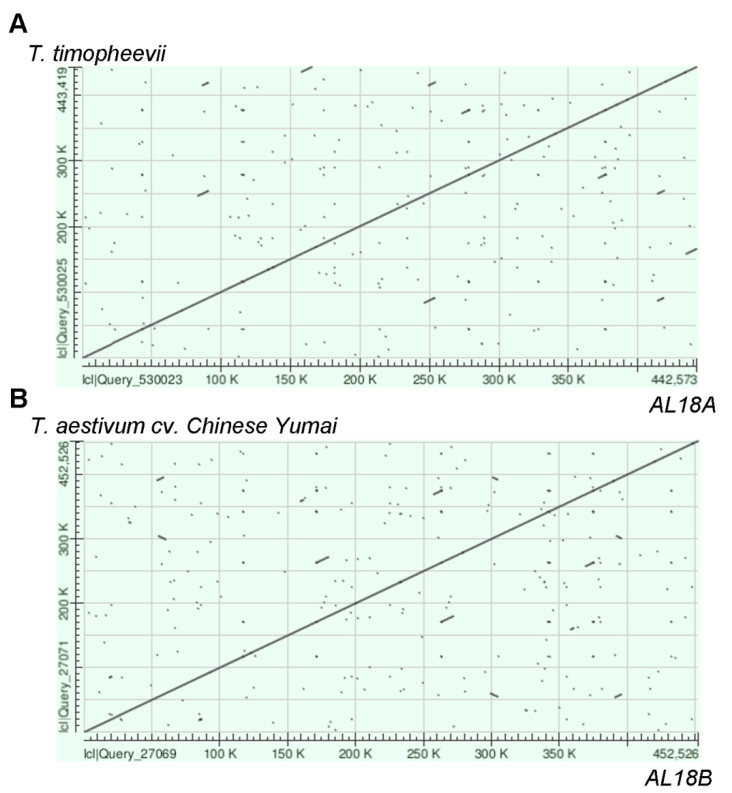
Comparison of mitochondrial DNA between AL18A and *Triticum timopheevii* (**A**), AL18B and Chinese Yumai (**B**). (**A**) The abscissa represents the mitochondrial DNA sequence of AL18A, and the ordinate represents the mitochondrial DNA sequence of *Triticum timopheevii*. (**B**) The abscissa represents the mitochondrial DNA sequence of AL18B, and the ordinate represents the mitochondrial DNA sequence of Chinese Yumai.

To explore the CMS genes in AL18A, we sequenced the mitochondrial genomes of AL18A and AL18B using Hiseq (next generation sequencing) and PacBio (single-molecule, real-time sequencing) sequencing technology. The Hiseq technology generated the clean data of 4116 MB and 5469 MB for AL18A and AL18B, respectively (Table 1). The length of subreads for AL18A ranged from 5000 to 15,000 bp, with an average of 7960 bp, and for AL18B, the read length ranged from 5000 to 10,000 bp, with an average length of 7467 bp, respectively (Table 2). After de novo assembly, the mitochondrial genome AL18A and AL18B were 442,712 and 452,491 bp in length, respectively. Mitochondrial DNA (mtDNA) sequence of AL18B was similar to *T. triticum* cv. Chinese Yumai (Figure 2B) and of AL18A was similar to *T. timopheevi* (Figure 2A), however, 102 SNPs and InDels were found between the later.

The flanking regions of the polymorphic sites were sequenced by sanger technology to validate the variations in the mitochondrial genome. As a result, the mitochondrial genome of AL18B was refined to 452,526 bp, consistent with that of Chinese Yumai, and AL18A was refined to 442,573 bp, similar but not identical to *T. timopheevi* (443,419 bp), respectively.

Fragmenting the mitochondrial genome of AL18A and *T. timopheevi* into two large sections, i.e., 1–21,521 bp and 21,428–442,573 bp for AL18A, and 1–21,518 bp and 22,310–443,419 bp for *T. timopheevi*, increased their sequence similarity up to 99%. These results predict that the mitochondria of the wheat AL-type CMS line may have derived from *T. timopheevi*, and the AL18A belongs to T-type CMS line.

### 2.3. The Mitochondrial Genome Composition of AL18A and AL18B

**Figure 3:**
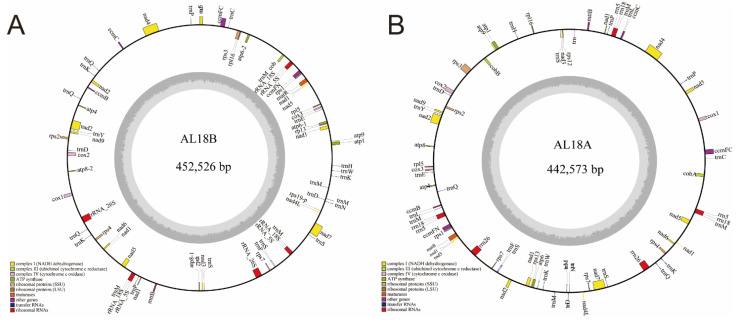
Circle map of AL18B (**A**) and AL18A (**B**) mitochondrial genome. Genes on the forward and reverse strands are presented on the outside and inside of the circle, respectively. The two gray layers represent the GC content in the mitochondrial genome.

The coding and non-coding genes of the verified mitochondrial genomes of AL18A and AL18B were predicted and assembled into a circular mitochondrial genome (Figure 3). The GC content of AL18A and AL18B was similar to the other wheat varieties, i.e., 44.34% and 44.35%, respectively. Comparative analysis of the three genomes revealed that AL18A and *T. timopheevi*, had same number of coding genes whereas, AL18B has two copies of *atp6* and *atp8*, and missing one copy of *ccmc-p*, *cobA*, *cox2a-p*, *rpl6-p*, and *rpl2-p* (Table 3). Analysis of the non-coding genes revealed that AL18A had four *Met-tRNA* and no *Ala-tRNA* while *T. timopheevi* had three *Met-tRNA* and one *Ala-tRNA*. In AL18B, there were two *Lys-tRNA* and two *Gln-tRNA*, but no *Leu-tRNA* and *Ile-tRNA*, whereas AL18A had two *Leu-tRNA* and one *Ile-tRNA* (Table 4).

### 2.4. CMS Candidate Genes of AL18A

**Figure 4:**
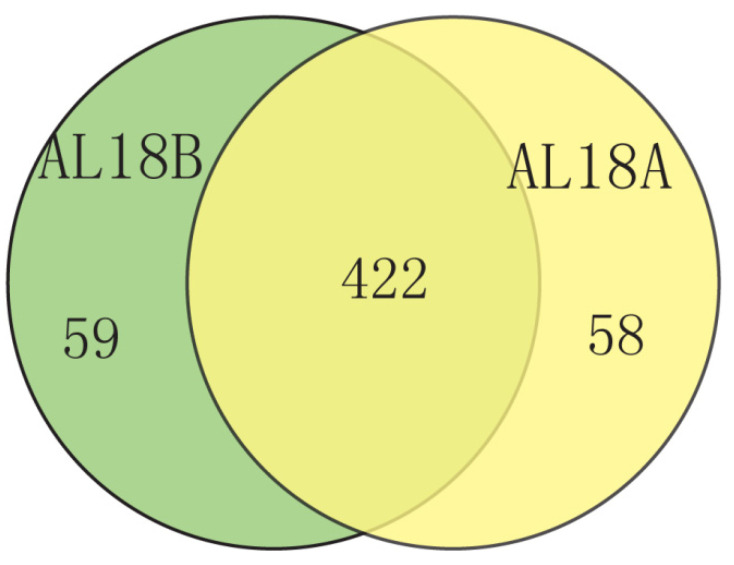
Venn diagram showing the mutual overlaps of *orfs* in the AL18B and AL18A.

Previously, it has been reported that mitochondrial CMS genes are produced by recombination of repetitive sequences, which have the characteristics of chimerism and co transcription [8,12]. Further, 523 and 526 *orfs*, longer than 200 bp, were predicted in the mitochondrial genomes of AL18A and AL18B, respectively. After removing the repetitive *orfs*, comparative analysis disclosed 58 *orfs* specific to AL18A (Figure 4). Transcriptomic analysis of 58 AL18A specific *orfs* revealed that 45 of them were explicitly expressed in the anther tissue of the male-sterile line and distributed on 13 locations of the AL18A mitochondrial genome. Trans-membrane structure analysis revealed that out of 45, only 11 *orfs* contained the transmembrane structure (Table 5), which hence were assumed to be the candidate CMS genes for AL18A.

**Figure 5:**
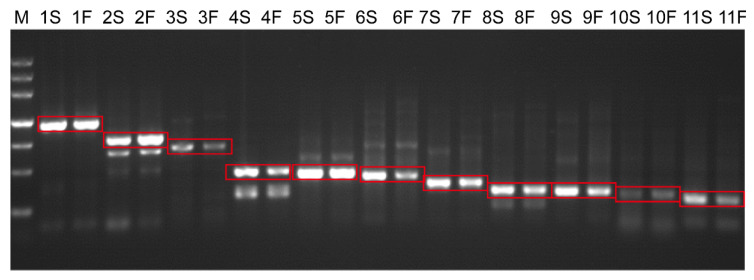
Transcription detection of 11 candidate *orfs* in AL18A and F_1_. M, molecular marker, from bottom to the upper, 200, 500, 800, 1200, 2000, 3000, 4500 bp, respectively; 1–11 represented 11 candidate *orfs* (*orf376*, *orf279*, *orf232*, *orf139*, *orf129*, *orf124*, *orf103*, *orf85*, *orf83*, *orf74*, and *orf66*) in AL18A and F_1_ (AL18A × 99AR144-1), S represents AL18A, and F represents F_1_ (AL18A × 99AR144-1).

Expression of candidate *orfs* were evaluated in the F_1_ hybrids of a cross between AL18A and a restorer line 99AR144-1 (AL18A × 99AR144-1). Results showed that all the candidate *orfs* were expressive in the presence of a restorer gene (Figure 5). As a female parent, AL18A was also crossed with wheat varieties Liangxing 99, Taishan 23, Zhongmai 895, Zhengmai 9045, and KN199, resulting in sterile F_1_ hybrids. These results indicated that the male parents did not contain the restorer gene, which hence could be used as AL18A maintainer lines. Nine out of 11 candidate genes (except *orf66* and *orf124*) were expressive in the F_1_ generations of all the five crosses.

It is reported that *orf256* might be the CMS gene in T-type CMS lines [17], which can also be the case for AL18A. Amplifying with PCR primers which located in the upstream of *orf256* and downstream of *cox1* of AL18A mitochondrial genome, produced two PCR bands. Sequencing and multiple alignment revealed the absence of *orf256* upstream of *cox1* in one of the sequences, while the other one was consistent with *T. timopheevi*, i.e., presence of *orf256* upstream of *cox1*. The chemical dose effect may cause this phenomenon in the plant mitochondrial genome, i.e., there were many kinds of mitochondrial DNA molecules in AL18A. In addition, the expression of *orf256* was also detected in AL18A by RT-PCR. Therefore, we considered *orf256* as a candidate gene, and its function was verified through the *Arabidopsis* transgenic system.

### 2.5. Functional Analysis of CMS Candidate Genes of AL18A

From AL18A, 12 candidate gene constructs were developed to study their function in the *Arabidopsis* transgenic system. The plant vector pCambia1300-221, driven by CaMV35S promoter and connected with a signal peptide from rice fertility restoring gene *Rf1b*, was used to target the mitochondria. Transgenic vectors were constructed both with 105 and 315 bp mitochondrial signal peptides, respectively.

The fertility of the transgenic plants containing the 315 bp of *Rf1b* as a mitochondrial signal peptide was not affected (Table 6). Additionally, 80% of the transgenic plants harboring the *orf66*, *orf83*, *orf85*, *orf103*, *orf124*, *orf129*, *orf139*, *orf256*, *orf232*, *orf279*, and *orf376* construct had more than 50% seed setting rate, implying these *orfs* has no role in sterility. The *orf74* construct significantly lowered fertility at the early developmental stages as 50% of the transgenics had less than 50% seed set; however, the fertility was gradually regained until maturity. Therefore, the function of *orf74* needed to be reconsidered.

**Figure 6:**
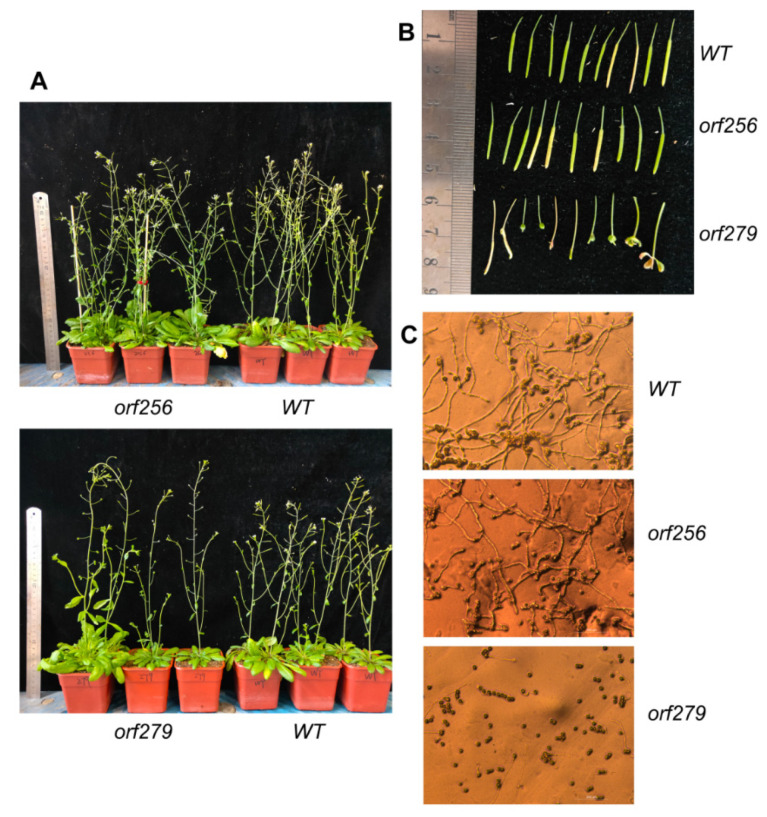
Phenotype of wild-type, *orf256* and *orf279* transgenic plants. (**A**) Transgenic plants; (**B**) siliques; (**C**) pollen germination rate, scale bar is 200 μm.

Fertility of the transgenic plants harboring the *orf279* and 105 bp of *Rf1b* as mitochondrial signal peptide were significantly reduced as 43% were almost sterile, 33% were partially fertile, and 24% were fully fertile (Table 7). Vegetative growth of transgenic plants was similar to wild-type plants; however, at the reproductive stage, the transgenic plants showed longer petiole, more prominent calyx, delayed floral degeneration, less pollen, lower pollen germination rate (Table 8, Figure S2), and abnormal pod development. The vegetative and reproductive growth of transgenic plants containing the *orf256*, previously considered as T-type CMS gene in wheat, was similar to that of wild-type plants, indicating that *orf256* is not related to sterility (Figure 6). Fertility of transgenics harboring *orf74*, *orf83*, *orf376*, *orf139*, *orf124*, *orf66*, *orf103*, *orf232*, *orf129*, and *orf83* was more than 87%, indicating these genes were not related to sterility.

**Figure 7:**
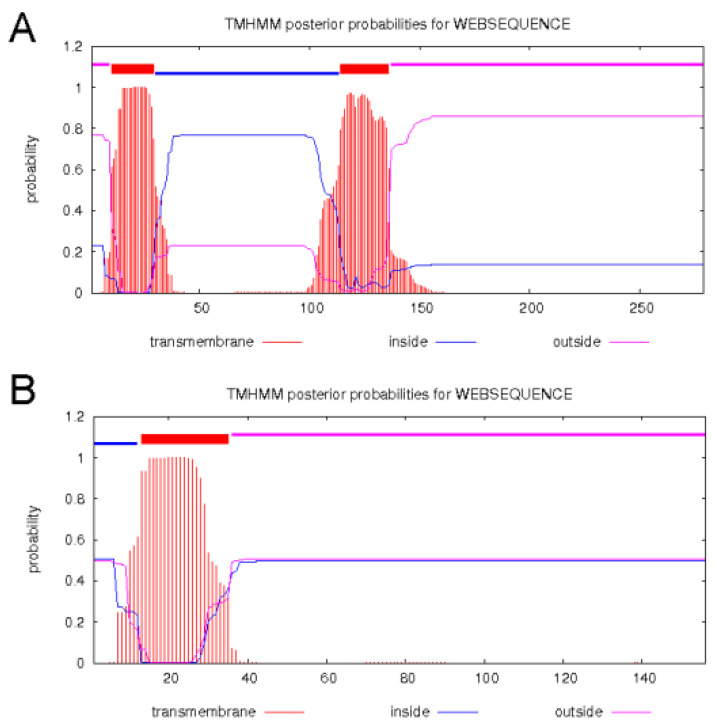
Transmembrane domain prediction of *orf279* (**A**) and *atp8* (**B**).

ORF279 contained two transmembrane domains (Figure 7A). Its function was annotated as an ATP synthase subunit 8 gene. ATP8 contained a transmembrane domain (Figure 7B). Compared to the protein sequences of ATP8 and ORF279, the first 96 amino acids of them were identical, and the first transmembrane domain of ORF279 was consistent with ATP8. It was speculated that ORF279 may affect the normal function of ATP8 and mitochondrial ATP synthesis.

## 3. Discussion

### 3.1. Mitochondrial Origin of AL-Type Cytoplasmic Male Sterile Line AL18A

In the 1990s, Wang et al. bred a cytoplasmic male sterile line AL18A [24]. Its cytoplasm was derived from a common wheat variety, ‘Huixianhong’, so they thought its mitochondrial genome should be similar to common wheat. By sequencing the mitochondrial genome of AL18A, we found that the mitochondrial genome of AL18A is almost identical to that of *T. timopheevi*, but significantly different from common wheat. Therefore, we believe that the mitochondrial genome of AL18A may be derived from *T. timopheevi* and belongs to T-CMS. There is some evidence that AL18A belongs to T-CMS. For example, Wang observed AL-type and other wheat CMS lines by scanning electron microscope and found that most mature pollen grains of AL-type male sterile lines were shrunk, and a few were plump, and the pollen sterility was 100%. Almost all the mature pollen grains of T-type male sterile lines were also shrunk, indicating that the AL-type and T-type CMS have similar pollen abortion phenotypes [24]. Jiang (2002) found that Q-type, T-type, and AL-type CMS lines of wheat have similar restoration and maintenance relationship, which may be the same kind of male sterile lines [25]. In conclusion, AL18A and T-CMS have the same morphology of sterile pollen grains, the same restoration and maintenance relationship, and a similar mitochondrial genome. Therefore, we conclude that AL18A belongs to T-CMS, and its mitochondria is from *T. timopheevi*.

### 3.2. The Male Sterile Gene of AL18A Is orf279

Early studies suggested that the candidate gene of T-CMS was *orf256*. The protein encoded by *orf256* existed in T-CMS line, and the expression of *orf256* changed in different nuclear backgrounds [16,26]. We also tried to verify the function of *orf256* in transgenic *Arabidopsis* and found that ORF256 had no role in sterility (Figure 6. However, *orf279* from the mitochondrial genome of AL18A led to male sterility in *Arabidopsis*, with longer petiole, more prominent calyx, delayed floral degeneration, shorter pod, lower pollen number, and germination rate. Recently, Australian scientists found that two fertility restoring genes *Rf1* and *Rf3* of T-CMS can bind to the mRNA of *orf279* in vitro and cleave to restore fertility. In contrast, the mRNA of *orf256* is not affected [19]. Therefore, *orf256* is not a CMS gene of T-CMS, while *orf279* might be the CMS gene of T-CMS.

### 3.3. Fertility Restoring Genes in 99AR144-1

The fertility restoring genes *Rf1* (Chr1A) and *Rf3* (Chr1B) bind to the mRNA of *orf279* and degrade it [19]. However, the expression of *orf279* in the F1 of AL18A × 99AR144-1 indicated that the restorer line 99AR144-1 harbored a different restorer gene. Liu (2010) reported that in 99AR144-1, fertility restoration was controlled by two major genes residing on Chr2A and Chr1B [27]. In contrast, the restorer gene *Rf1* and *Rf3* are on Chr1A and Chr1B, respectively, indicating a different restorer gene in 99AR144-1.

### 3.4. Tapetal PCD and Male Sterility

The timely PCD of tapetum is very important for pollen development [28]. Luo (2013) reported that the abortion of WA-CMS in rice was caused by the advance of PCD in tapetum. The male-sterile protein WA352 inhibits the scavenging effect of COX11 on reactive oxygen species, resulting in the explosion of ROS in mitochondria and the release of cytochrome C. The cytochrome C eventually led to the advanced programmed cell death in tapetum, resulting in the abnormal nutrient absorption of pollen grains and its abortion [3]. In this study, we found that the earliest abnormal period of AL18A anther development compared with AL18B was the uni-nucleate stage, which showed delayed cell collapse in tapetum, indicating a delayed PCD in the tapetum. Further research is needed to understand the relationship between delayed PCD and ROS level and the effect of ROS in tapetum through a specific mechanism.

### 3.5. The Importance of Mitochondrial Signal Peptide in Functional Verification of CMS Gene

In this study, the first 315 and 105 bp of *Rf1b* from rice were used as mitochondrial targeting signal peptides. The transgenic *Arabidopsis* of *orf279* with 105 bp of signal peptide showed evident sterility. *Rf1b* is a PPR restorer gene that has 506 amino acids. The first 315 bp of *Rf1b*, encoding the first 23 amino acids as the putative mitochondrial signal peptide, the first PPR repeat (53–81 amino acids), and part of the second PPR repeat (92–105 amino acids), may affect the function of ORF279 protein. However, the first 105 bp of *Rf1b* only encodes the putative signal peptide sequence and does not contain the PPR repeat sequence, so it may target the mitochondria without affecting the function of ORF279.

## 4. Materials and Methods

### 4.1. Plant Materials

Wheat (*Triticum aestivum* L.) cytoplasmic male sterile line AL18A, maintainer line AL18B, and a restorer line 99AR144-1 were kindly provided by professor Xiaoming Tian of the Xinjiang Academy of Agricultural Reclamation Sciences. Other wheat cultivars (KN199, Zhongmai 895, Taishan 23, and Liangxing 99) and *Arabidopsis thaliana* (Col-0) were provided by the State Key Laboratory of Plant Cell and Chromosomal Engineering.

### 4.2. Scanning of Wheat Anthers Using a Scanning Electron Microscope

Anther and pollen samples of AL18A and AL18B were prepared according to Nguyen and Harbison (2017) and scanned with a scanning electron microscope (Hitachi S-3000N & Quorum PP3000T) [29].

### 4.3. Extraction of Plant Mitochondrial DNA

Two-weeks-old, etiolated seedlings of AL18A and AL18B were used to isolate the mitochondria following the published protocol [20,30]. Mitochondrial fractions were collected by differential centrifugation and incubated with DNase I for one hour in ice to eliminate the linear DNA. The mitochondrial samples further purified by discontinuous sucrose density gradient centrifugation (1.2 M/1.6 M/2.0 M) were carefully collected from the 1.6 M/1.2 M interface and washed with 0.4 M sucrose solution. The fraction was finally lysed in 2% Sarkosyl for mitochondrial DNA (mtDNA) extraction, followed by phenol-chloroform extraction and ethanol precipitation.

### 4.4. Mitochondrial Genome Sequencing Analysis

The mtDNA of AL18A and AL18B (accession number MW846284 and MW846283, respectively) were sequenced at Mega genomics Co. LTD, and the sequenced reads were assembled using the software package SOAPdenovo (v2.04)/SPAdes-3.10.1 on a PC/UNIX platform. The mitochondrial sequences were annotated with AUGUSTUS (http://bioinf.uni-greifswald.de/augustus/, accessed on 22 May 2021) and tRNA genes and their secondary structures were identified using RNAmmer-1.2 and tRNAscan-SEv1.3.1. The Pairwise BLAST program on our local server was used for comparison between AL18A mtDNA and AL18B mtDNA and the mitochondrial genome of *Triticum timopheevii*, with an E-value cutoff at 0.001. A database search was executed using the nucleotide BLAST network service (https://blast.ncbi.nlm.nih.gov/Blast.cgi?PROGRAM=blastn&PAGE_TYPE=BlastSearch&LINK_LOC=blasthome, accessed on 22 May 2021) with default parameters. Alignments were obtained using MultiPipMaker, a web-based tool for genomic sequence alignments (http://bio.cse.psu.edu/pipmaker, accessed on 22 May 2021) [31,32]. The annotated AL18A mtDNA genomic sequence was used as a reference genome and compared with mtDNA sequences from AL18B (*T. aestivum*).

### 4.5. Vector Construction and Genetic Transformation of Arabidopsis thaliana

The PCR primers with a restriction site overhang were designed from the conserved domain region of the target genes. The PCR products were transformed into *E. coli* after being ligated into pCambia1300-121 vector by double restriction enzyme digestion and the positive clones were screened on Kanamycin selectable LB plates. The positive constructs were transformed in *Arabidopsis* (Col-0) using the floral dip method [33]. T_0_ seeds were screened on a selectable (Hygromycin) ½ MS media. After two weeks, the transgenic seedlings were shifted in soil medium (1:1) and grown at 23 °C, 16:8 h photoperiod until maturity.

### 4.6. Pollen Germination Experiment of Arabidopsis thaliana

The pollen germination of *Arabidopsis thaliana* was studied according to Hirsche et al. (2017) [34].

## Figures and Tables

**Table 1:** Illumina PE sequencing data statistics of AL18A and AL18B.

Sample ID	Insert Size (bp)	Raw Data (Mb)	Clean Data (Mb)	Reads Length (bp)	Clean Data GC (%)	Clean Data Q20 (%)	Clean Data Q30 (%)
AL18A	450	4325	4116	(150:150)	48.15	97.75	93.22
AL18B	430	5806	5469	(150:150)	45.82	98.59	95.45

**Table 2:** PacBio sequencing data statistics of AL18A and AL18B.

Sample ID	Subreads Number	Subreads Bases (bp)	Subreads Largest Length (bp)	Subreads N50 Length (bp)	Subreads N90 Length (bp)	Subreads Average Length (bp)
AL18A	10,307	82,044,853	21,253	9924	5003	7960
AL18B	44,802	134,889,953	21,302	7467	909	3011

**Table 3:** Comparison of coding genes in different mitochondrial genomes.

Functional Category	AL18A	*Triticum timopheevii*	AL18B	Functional Category	AL18A	*Triticum timopheevii*	AL18B
Complex V	*atp1*	*atp1*	*atp1*	Complex I	*nad1*	*nad1*	*nad1*
	*atp4*	*atp4*	*atp4*		*nad2*	*nad2*	*nad2*
	*atp6*	*atp6*	*atp6-1*		*nad3*	*nad3*	*nad3*
			*atp6-2*		*nad4*	*nad4*	*nad4*
	*atp8*	*atp8-1*	*atp8-1*		*nad4L*	*nad4L*	*nad4L*
	*atp8-p*	*atp8-p*	*atp8-2*		*nad5*	*nad5*	*nad5*
	*atp9*	*atp9*	*atp9*		*nad6*	*nad6*	*nad6*
Cytochtome c biogenesis	*ccmB*	*ccmB*	*ccmB*		*nad7*	*nad7*	*nad7*
	*ccmC-1*	*ccmC-1*	*ccmC*		*nad9*	*nad9*	*nad9*
	*ccmC-p*	*ccmC-p*		Ribosomal proteins	*rpl16*	*rpl16*	*rpl16*
	*ccmFC*	*ccmFC*	*ccmFC*		*rpl16-p*	*rpl16-p*	
	*ccmFN*	*ccmFN*	*ccmFN*		*rpl2-p*	*rpl2-p*	
Complex III & IV	*cobA*	*cobA*			*rpl5*	*rpl5*	*rpl5*
	*cobB*	*cobB*	*cobB*		*rps1*	*rps1*	*rps1*
	*cox1*	*cox1*	*cox1*		*rps12*	*rps12*	*rps12*
	*cox2*	*cox2*	*cox2*		*rps13*	*rps13*	*rp13*
	*cox2a-p*	*cox2a-p*			*rps19-p*	*rps19-p*	*rps19-p*
	*cox3*	*cox3*	*cox3*		*rps2*	*rps2*	*rps2*
others	*matR*	*matR*	*matR*		*rps3*	*rps3*	*rps3*
	*mttB*	*mttB*	*mttB*		*rps4*	*rps4*	*rps4*
					*rps7*	*rps7*	*rps7*

**Table 4:** Comparison of noncoding genes in different mitochondrial genomes.

Functional Category	AL18A	*Triticum timopheevii*	AL18B	Functional Category	AL18A	*Triticum timopheevii*	AL18B
rRNAs	*rrn18-1*	*rrn18-1*	*rrn18-1*	tRNA (amino acid)	Asn	Asn	Asn
	*rrn18-2*	*rrn18-2*	*rrn18-2*		Met	Met	Met
	*rrn18-3*	*rrn18-3*	*rrn18-3*		Trp	Trp	Trp
	*rrn26-1*	*rrn26-1*	*rrn26-1*		Met	Met	Met
	*rrn26-2*	*rrn26-2*	*rrn26-2*		Asp	Asp	Asp
			*rrn26-p*		Met	Met	Met
	*rrn5-1*	*rrn5-1*	*rrn5-1*		Ser	Ser	Ser
	*rrn5-2*	*rrn5-2*	*rrn5-2*		Phe	Phe	Phe
	*rrn5-3*	*rrn5-3*	*rrn5-3*		Ser	Ser	Ser
tRNAs (amino acid)	Cys	Cys	Cys		Asp	Asp	Asp
	Pro	Pro	Pro		Tyr	Tyr	Tyr
	Gln	Gln	Gln		His	His	His
	Lys	Lys	Lys		Met		Met
	Gln	Gln	Gln				Lys
	Glu	Glu	Glu				Gln
	Lys	Lys	Lys		Leu	Leu	
	Met	Met	Met		Leu	Leu	
	Pro	Pro	Pro			Ala	
	Ser	Ser	Ser		Ile	Ile	

**Table 5:** Description of eleven *orfs* containing transmembrane domains in AL18A.

Number	*orf*	Start	End	Direction	Size (bp)	Description
1	*orf376*	170,465	171,595	+	1131	Ribosomal protein S2
2	*orf279*	111,818	112,657	−	840	ATP synthase subunit 8
3	*orf232*	296,072	296,770	−	699	
4	*orf139*	295,683	296,102	−	420	Hypothetical protein
5	*orf129*	112,174	112,563	−	390	*orf143* (mitochondrion)
6	*orf124*	156,900	157,274	−	375	
7	*orf103*	207,567	207,878	+	312	
8	*orf85*	156,879	157,136	+	258	
9	*orf83*	296,477	296,728	+	252	
10	*orf74*	25,285	25,509	+	225	
11	*orf66*	23,773	23,973	+	201	

**Table 6:** Phenotypic study of transgenic *Arabidopsis thaliana* containing 315 bp mitochondrial signal peptide of *Rf1b*.

Candidate *orfs*	Number of T1 Plants	Seed Setting Rate of T1 Plant (%)			
		>90	51–90	11–50	1–10
CK-Rf1b	9	9 (100%)			
*orf66*	32	26 (81%)		4 (13%)	2 (6%)
*orf74*	28	8 (29%)	6 (21%)	7 (25%)	7 (25%)
*orf85*	26	24 (92%)	1 (4%)		1 (4%)
*orf103*	51	39 (76%)	2 (4%)	5 (10%)	5 (10%)
*orf279*	49	39 (80%)	2 (4%)	6 (12%)	2 (4%)
*orf129*	38	37 (97%)			1 (3%)
*orf139*	37	32 (87%)		2 (5%)	3 (8%)
*orf83*	36	33 (92%)			3 (8%)
*orf232*	14	14 (100%)			
*orf124*	16	13 (82%)		1 (6%)	2 (12%)
*orf376*	67	64 (96%)		2 (3%)	1 (1%)
*orf256*	57	55 (96%)			2 (4%)

**Table 7:** Phenotypic study of transgenic *Arabidopsis thaliana* containing 105 bp mitochondrial signal peptide of *Rf1b*.

Candidate *orfs*	Number of T1 Plants	Seed Setting Rate of T1 Plant (%)		
		1–50	51–90	>90
*orf279*	61	26 (43%)	20 (33%)	15 (24%)
*orf74*	36	1 (3%)	2 (6%)	33 (91%)
*orf83*	36			36 (100%)
*orf256*	57	3 (5%)		54 (95%)
*orf376*	53	3 (6%)	3 (6%)	47 (88%)
*orf139*	28			28 (100%)
*orf124*	36			36 (100%)
*orf66*	53	1 (2%)	2 (4%)	50 (94%)
*orf103*	60	1 (2%)	1 (2%)	58 (96%)
*orf232*	23	1 (4%)	2 (9%)	20 (87%)
*orf129*	19	1 (5%)		18 (95%)
*orf85*	32	2 (6%)		30 (94%)

**Table 8:** Pollen germination rate of transgenic plants.

Candidate *orfs*	Flower Number of T1 Plants	Pollen Germination Rate (%)		
		<30	30–60	>60
*orf279*	122	50 (41%)	38 (31%)	34 (28%)
*orf74*	72	2 (3%)	4 (6%)	66 (91%)
*orf83*	72		2 (3%)	70 (97%)
*orf256*	114	6 (5%)	2 (2%)	106 (93%)

## Data Availability

The datasets used and/or analyzed during the current study are available from the corresponding author on reasonable request.

## Supplementary Materials

The following are available online at https://www.mdpi.com/article/10.3390/ijms22126388/s1, Figure S1: Seeds setting of AL18A, AL18B and F_1_ (AL18A × AL18B), Figure S2: Pollen germination rate of WT, transgenic plants of *orf256* and *orf279*, respectively.
