# Supplementary material for: Characterization of the Mitochondrial Genome of a Wheat AL-Type Male Sterility Line and the Candidate CMS Gene
Source: Int J Mol Sci. 2021 Jun 15;22(12):6388. doi: 10.3390/ijms22126388 (PMC8232308; doi:10.3390/ijms22126388)
Supplement: Supplementary file 1 [file ijms-22-06388-s001.zip › ijms-1250893-supplementary.pdf]

International Journal of
Molecular Sciences

MDPI

Article

Characterization of the mitochondrial genome of a wheat AL-type male sterility line and the candidate CMS gene

Miaomiao Hao [1,2,†], Wenlong Yang [1,3,†,*], Weiwen Lu [1,2], Linhe Sun [1,2,4], Muhammad Shoaib [1,2], Jiazhu Sun [1], Dong-cheng Liu [1], Xin Li [1] and Aimin Zhang [1,*]

[1] State Key Laboratory of Plant Cell and Chromosome Engineering, Institute of Genetics and Developmental Biology/Innovative Academy of Seed Design, Chinese Academy of Sciences, Beijing, 100101, China;
[2] University of Chinese Academy of Sciences, Beijing, 100049, China;
[3] Institute of Vegetables and Flowers, Chinese Academy of Agricultural Sciences, Beijing, 100081, China;
[4] Institute of Botany, Jiangsu Province and Chinese Academy of Sciences (Nanjing Botanical Garden Mem. Sun Yat-Sen), Nanjing, 210014, China;
† These authors contributed equally to this work.
* Correspondence: Aimin Zhang (amzhang@genetics.ac.cn, 008610-64806618), Wenlong Yang (wlyang@genetics.ac.cn, 008610-64806617)

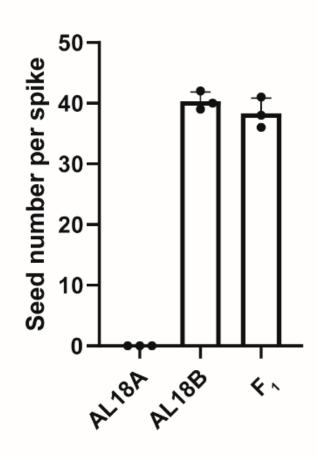

Figure S1. Seeds setting of AL18A, AL18B and F_1 (AL18A × AL18B).

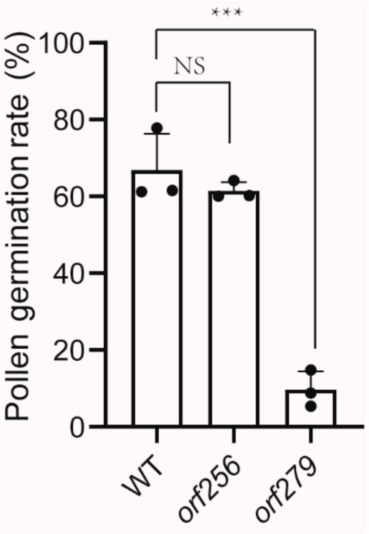

Figure S2. Pollen germination rate of WT, transgenic plants of *orf256* and *orf279*, respectively. Student *t*-test was used for pollen germination rate comparison between wild-type (WT) *Arabidopsis* and transgenic plants of *orf256* and *orf279* individually. NS (P = 0.3929), non-significant difference. ***P = 0.0007. Data are means ± S.D. n = 3 independent plants.
